# BDNF Increases Survival and Neuronal Differentiation of Human Neural Precursor Cells Cotransplanted with a Nanofiber Gel to the Auditory Nerve in a Rat Model of Neuronal Damage

**DOI:** 10.1155/2014/356415

**Published:** 2014-08-26

**Authors:** Yu Jiao, Björn Palmgren, Ekaterina Novozhilova, Ulrica Englund Johansson, Anne L. Spieles-Engemann, Ajay Kale, Samuel I. Stupp, Petri Olivius

**Affiliations:** ^1^Center for Hearing and Communication Research, Karolinska University Hospital, 171 76 Stockholm, Sweden; ^2^Department of Clinical Sciences, Intervention and Technology (CLINTEC), Section of Otorhinolaryngology, Karolinska Institutet, Karolinska University Hospital, 171 76 Stockholm, Sweden; ^3^Department of Otolaryngology, Head and Neck Surgery, Beijing Tongren Hospital, Capital Medical University, Beijing 100730, China; ^4^Department of Ophthalmology, Institution of Clinical Sciences in Lund, Lund University, 221 84 Lund, Sweden; ^5^Departments of Chemistry, Materials Science and Engineering, and Medicine and Institute for Bionanotechnology in Medicine, Northwestern University, Chicago, IL 60208, USA; ^6^Division of Otorhinolaryngology, Linköping University Hospital, 581 85 Linköping, Sweden; ^7^Division of Otorhinolaryngology, Department of Clinical and Experimental Medicine, University of Linköping, 581 85 Linköping, Sweden

## Abstract

*Objectives*. To study possible nerve regeneration of a damaged auditory nerve by the use of stem cell transplantation. *Methods*. We transplanted HNPCs to the rat AN trunk by the internal auditory meatus (IAM). Furthermore, we studied if addition of BDNF affects survival and phenotypic differentiation of the grafted HNPCs. A bioactive nanofiber gel (PA gel), in selected groups mixed with BDNF, was applied close to the implanted cells. Before transplantation, all rats had been deafened by a round window niche application of *β*-bungarotoxin. This neurotoxin causes a selective toxic destruction of the AN while keeping the hair cells intact. *Results*. Overall, HNPCs survived well for up to six weeks in all groups. However, transplants receiving the BDNF-containing PA gel demonstrated significantly higher numbers of HNPCs and neuronal differentiation. At six weeks, a majority of the HNPCs had migrated into the brain stem and differentiated. Differentiated human cells as well as neurites were observed in the vicinity of the cochlear nucleus. *Conclusion*. Our results indicate that human neural precursor cells (HNPC) integration with host tissue benefits from additional brain derived neurotrophic factor (BDNF) treatment and that these cells appear to be good candidates for further regenerative studies on the auditory nerve (AN).

## 1. Introduction

Although stem cell therapy in many ways is still in its infancy for treating neurodegenerative diseases or nerve trauma, cell transplantation provides a promising strategy for treatment of several lesions in the central nervous system (CNS). In humans, degeneration of the auditory nerve (AN) in the inner ear secondary to hair cell loss is an irreversible process, which eventually reduces residual hearing in any hearing impaired patient [1, 2]. Additionally, there are a number of lesions and diseases that primarily affect the AN, inducing profound to total hearing loss. Such lesions include tumors (most commonly caused by neurofibromatosis type 2), surgery, trauma, and auditory neuropathy [3]. Such central hearing impairment cannot be improved by hearing aids or cochlear implants (CI), since these are designed to aid patients with a cochlear sensorineural hearing loss illustrating that these are dependent on a functioning AN. For example, it has been demonstrated that the success of a CI is dependent on the proper transduction of electrical signals and subsequently on the function of the AN [4, 5]. Auditory brain stem implants (ABI) have been developed as one possible treatment for central hearing loss (i.e., along the AN). Even though several studies have reported that for some patients excellent speech understanding is possible with an ABI [6, 7], other results obtained from such implants have only improved speech recognition as compared with lip reading [8]. Thus, there is a need for an improved treatment strategy for hearing loss caused by permanently malfunctioning or lesioned spiral ganglion neurons (SGNs). The AN is composed of bipolar SGNs with peripheral processes and central axons and is located in Rosenthal's canal within the modiolus in the cochlea. The SGNs constitute the afferent innervation from the hair cells and transduce the nerve impulses to the next relay station in the cochlear nucleus.

A theoretical method of restoring hearing for patients with a permanent neuronal hearing loss may be to regenerate the injured auditory pathway with stem cells. Neural stem cells are precursor cells with the ability to differentiate into neurons and glial cells. These can be not only derived from embryonic stem cells (ESCs) but also found within the adult nervous system [9].* In vitro*, we have demonstrated the capability of several different cell types to integrate into the cochlear nucleus, being the second order neuron in the auditory neuronal pathway [10–12]. Furthermore, in a previous* in vivo* study we demonstrated good survival and differentiation of mouse tau-green fluorescent protein (GFP) embryonic stem cells transplanted to the AN in deafened rats [13]. However, good survival, differentiation, and, most importantly, functional improvement of hearing are still challenges for any* in vivo* experiment with stem cells transplanted to the AN. To achieve a properly restored neuronal circuitry, the transplanted cells need to differentiate into neurons and display regulated outgrowth of axons and dendrites with accurate target selection [14].

Human neural precursor cells (HNPCs) have in several studies proved an excellent capacity to generate neurons after* in vivo* intracerebral transplantation [15–17]. Such cells have been studied in animal models of neural degenerative diseases such as Huntington's and Parkinson's diseases with a remarkable ability to form site-specific neurons and to some extent have a functional effect [17–20]. In addition, the possibility to culture human spiral ganglion tissue to obtain neurons indicates a presence of HNPCs also in the adult human auditory nervous system [21]. Encouragingly, a recent report describes a protocol to induce differentiation of human ESCs into otic neuroprogenitors. The differentiated cells were subsequently implanted into a gerbil auditory neuropathy model [22]. Significantly improved auditory-evoked response thresholds were detected for up to 10 weeks after implantation indicating functional recovery.

Neurotrophic factors are important for the development and maintenance of SGNs. For example, exogenous brain derived neurotrophic factor (BDNF) has in* in vitro* as well as* in vivo* studies proved to promote SGN survival [23–25]. It has further been shown that ESCs express the tropomyosin-related kinase (TRK) receptors that mediate antiapoptotic signals, including the BDNF receptor trkB [26]. Thus, a more beneficial approach of regeneration or enhancement of the AN may be to use a combination of transplanted cells and neurotrophic factors.

Tissue engineering is an approaching research field, and numerous studies are describing the advantages with using nanomaterials for improving graft-host integration [27]. Our laboratory has studied a bioactive nanofiber gel consisting of self-assembling peptide amphiphile (PA) molecules designed to present the neurite-promoting laminin epitope isoleucine-lysine-valine-alanine-valine (IKVAV) to the transplanted cells [28]. When mouse-derived neural precursor cells are encapsulated* in vitro* in these gels, rapid and selective differentiation of the cells into neurons is observed [28]. Furthermore, the PA gel has also been shown to have an inhibitory effect on astrocytes, thus preventing scar formations [29]. In an earlier study we have shown a beneficial effect of BDNF and PA gel on mouse tau-GFP cells injected to the internal auditory meatus (IAM) or the modiolus [13].

Here, we used a previously established rodent model of selective AN lesion [30] and injected HNPCs either with the PA gel only or with BDNF added in the PA gel. In order to cause minimal damage to the donor site, due to the limited space in the thin AN, the PA gel was applied over the injection site subsequent to the deposition of the HNPCs. HNPCs were carrying the reporter gene GFP. Previously, GFP expression has been found long-term in neurons as well as in glial cells after transplantation of similar HNPCs [15, 31]. The GFP distribution found throughout the entire cytoplasm and fine structures of the HNPCs allowed for detailed morphological analysis.

In the present study we demonstrate the beneficial effect of BDNF contained in the PA gel. This treatment rendered a significant larger number of cells, increased neuronal differentiation, and migration of the GFP+ HNPCs after injection into the AN. Furthermore, histological analysis demonstrated neurite outgrowths and arborisation in the transplanted HNPCs as well as fiber growth into the cochlear nucleus area of the brain stem (BS).

## 2. Materials and Methods

### 2.1. Animals

All animal experiments followed the national approved protocol for care and use of animals in Sweden (N3/11; N4/11). Young adult female Sprague-Dawley rats (*n* = 13; 200–250 g) were used in this study. To exclude any visible middle ear infections, preoperative otoscopic examinations were performed.

### 2.2. Application of *β*-Bungarotoxin to the Round Window Niche

Three weeks prior to HNPC injection, the animals (*n* = 13) were deafened by application of *β*-bungarotoxin (*β*-BuTx) to the round window niche as previously described [30]. In brief, after anesthesia with xylazine (10 mg/kg i.p.) and ketamine (50 mg/kg i.p.), the round window niche was exposed by a retroauricular incision. Five microliters of *β*-BuTx (0.05 *μ*g/mL, Alexis Biochemicals) was absorbed by gel foam and applied to completely fill the round window niche. A piece of fascia was placed to cover the hole in the bulla and the wound was sutured.

### 2.3. Human Neural Precursors (HNPCs) Cell Line

The human neural precursor cell line used for this study was originally established by L. Wahlberg, Å. Seiger, and colleagues at the Karolinska University Hospital (original work with the cell line was described by Carpenter et al. [32] and was kindly provided by Professor A. Björklund (Department of Experimental Medical Science, Lund University Sweden)). Briefly, forebrain tissue was obtained from a nine-week-old (postconception) human embryo and isolated under compliance with the National Institute of Health guidelines, Swedish Government guidelines, and the local ethical committee. The HNPCs were cultured in DMEM-F12 medium (Invitrogen) supplemented with 2.0 mM L-glutamine (Sigma), 0.6% glucose (Sigma), N2 supplement (Invitrogen), and 2.0 mg/mL heparin (Sigma) and were cultured as free floating clusters (neurospheres). The growth factors: human basic fibroblast growth factor (hbFGF, 20 ng/mL; R&D Systems), human epidermal growth factor (hEGF, 20 ng/mL; Invitrogen), and human leukemia inhibitory factor (hLIF, 20 ng/mL; Sigma), were added every 3–5 days to the culture. The neurospheres were passaged by mechanical dissociation every 7–10 days and reseeded as single cells at a density of 1 × 10^5^ cells/mL. HNPCs expressed the reporter gene GFP which was previously transduced to the cells using a lentiviral infection at MOI = 0,1 (for details on lentiviral infection see [33]).

### 2.4. Surgical Approach

**Figure 1:**
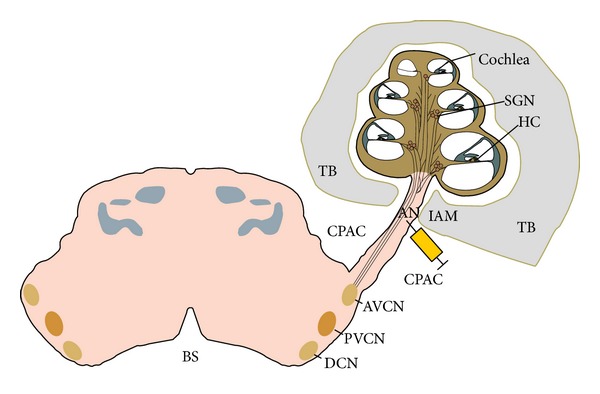
Schematic of the cochlea, the auditory nerve, and the brain stem. The syringe indicates the HNPC injection site in the auditory nerve trunk by the internal auditory meatus. AN: auditory nerve; TB: temporal bone; CPAC: cerbellopontine angle cistern; IAM: internal auditory meatus; HC: hair cell; SGN: spiral ganglion neuron; AVCN: anteroventral cochlear nucleus; PVCN: posteroventral cochlear nucleus; DCN: dorsal cochlear nucleus; BS: brain stem.

The surgical approach for cell injections (C.Inj.) into the AN trunk by the IAM (Figure 1) has been previously described [34]. In brief, all animals were anaesthetized with an intraperitoneal (i.p.) injection of a mixture of Ketalar (50 mg/kg) and Rompun (10 mg/kg) and the skull was put in a fixed position with the aid of a stereotactic frame. Under a surgical microscope, an incision was made through the skin and underlying soft tissue. Using a drill, a hole was made in the suboccipital bone and the underlying dura was opened and reflected towards the edge of the hole. The cerebellar hemisphere was retracted medially to reveal the AN entering through the IAM.

### 2.5. Cell Injections

A microsyringe mounted in the clamping device of the stereotactic frame was used for all C.Injs. The needle was positioned above the AN trunk with the angle of the tip adjusted towards the IAM. The needle was lowered into the AN trunk by the use of a micromanipulator and 5 *μ*L of HNPCs dissociated into a single cell suspension in culture medium (10.000 cells/*μ*L, i.e., a total of 50.000 cells/implant) was injected over one minute. After injection, the needle was left in place for 10 minutes. The wound cavity was filled with sterile saline and a piece of fascia was applied to cover the hole in the dura and occipital bone. The wound was sutured in layers. Following surgery and removal from the stereotactic frame, the animals were given subcutaneous injections of 3 mL saline and 0.2 mL Temgesic (0.3 mg/mL) and placed in a warm cage to recover before being transferred to their home cage.

To prevent postoperative infection and immune response rejection, all animals received daily doses of tetracycline (1.8 mg/mL, i.p.) and cyclosporine (4.2 mg/mL, i.p.) until sacrificed.

### 2.6. BDNF and PA Gel Applications

In Groups 1–3, 1 wt% IKVAV peptide amphiphile nanofiber gel (Nanotope, USA) was applied over the C.Inj. site by the IAM in the same surgical session as the C.Inj. In two groups (Groups 2 and 3) an additional 10 *μ*L high concentration BDNF (20 mg/mL) was mixed with the applied PA gel.

### 2.7. Experimental Groups

The animal groups are described in Table 1. In Groups 1–3 the HNPCs were injected into the left AN trunk near the IAM in the central portion of the AN (Figure 1). Group 4 served as the control group with culture medium (vehicle) only injected.

### 2.8. Tissue Preparation

After three or six weeks of survival, rats were sacrificed by an overdose of pentobarbital (60 mg/mL, i.p.) and transcardially perfused with body warm 0.9% saline followed by ice-cold 4% paraformaldehyde (PFA) in 0.1 M phosphate buffer saline (PBS). The cochlea, the AN, and part of the BS were carefully removed from the temporal bone en bloc. The specimens (cochlea plus AN including BS) were further dissected and a small hole was made in the apex of the cochlea through which the cochlea was perfused with PFA (initially with 4% and then with 0.5%). The cochlea was decalcified for seven days using 1% ethylenediaminetetraacetic acid in 0.1 M PBS. After decalcification, the specimens spent 24 hours in 20% sucrose solution and were embedded and frozen in Optimal Cutting Temperature Compound (Tissue-Tek; Sakura Finetek, Torrance, CA, USA). The specimens were orientated in the compound so that the midmodiolar sections would contain the cochlea, the AN, and the BS as a continuum. The 12 *μ*m midmodiolar cryosections were mounted on glass slides.

### 2.9. Immunohistochemistry

Sections were fixed in 4% PFA in phosphate buffered saline (PBS, pH 7.4) for 1 h at room temperature (RT). After washing 3 times in PBS, the sections were treated with ice cold 20% methanol in PBS for 5 min at RT. After 3 more rinses in PBS, the tissue was permeabilized using 0.5% Triton-X in PBS overnight at 4°C. The sections were washed 3 times with PBS and incubated with 20% bovine serum albumin (Sigma) blocking solution for 12 h at 4°C prior to incubation with primary antibodies. The sections were incubated at 4°C overnight with fluorescein isothiocyanate conjugated goat polyclonal GFP antibody for transplanted cell detection (1 : 200 dilution; Abcam, Cambridge, UK). For double immunostaining, the sections were incubated at 4°C overnight with a primary rabbit polyclonal *β*-tubulin (TUJ1) antibody (1 : 200 dilution; Covance Research Products, Berkeley, CA, USA). Following incubation with the primary antibodies, the sections were incubated with secondary goat-anti-rabbit Cy3 antibody (1 : 2000 dilution; Jackson Immuno Research) for four hours at RT. Omission of the primary antibody served as negative control. Cell nuclei were stained with 4,6-diamidino-2-phenylindole (DAPI). After three more rinses with PBS the sections were mounted with Prolong Gold mounting medium (Invitrogen) and examined using a fluorescence microscope (Axio Observer Z1, Zeiss). The brightness and contrast of the presented images were adjusted to aid visualization (Adobe Photoshop CS5 12.0, Adobe Systems Inc., USA).

### 2.10. Quantification of Transplanted and Differentiated Cells

The specimens were oriented and cryosectioned with the cochlea, the AN, and the BS in the same section. Surviving transplanted cells were defined as cell profiles with coexpression of GFP and DAPI. Differentiated transplanted cells were defined as cell profiles with coexpression of GFP and TUJ1. Quantification and statistical analysis were performed on all groups assessing the number of GFP- and DAPI-stained cells and GFP- and TUJ1 double-labeled cells. We further quantified the number of differentiated HNPCs with nerve fiber outgrowths.

From a total of approximately 30 serial sections from each specimen, the number of HNPCs was in quantified in four regions. These regions include the scala tympani (ST) in the cochlea, the modiolus in the cochlea, the AN, and the BS including the cochlear nucleus. The restrictions of the regions were set in the microscope in accordance with histological characteristics (e.g., the transitional zone between the AN and modiolus). Quantification was performed using stereology, as previously described [13, 35].

Throughout the entire specimen, quantification was performed in every third section. Since the average diameter of the profiles quantified was 15 *μ*m and the sections had a thickness of 12 *μ*m, this avoided double counting of profiles. In every analyzed section, cells were quantified in each of the four different regions individually. Every third section of a region was subdivided into fields where each field was equivalent to the microscopic optic field at 40x. In order to get a mean value of cells per section for each region, the total number of counted cells in each region was divided by the number of evaluated sections. Further, for every region, the mean number of cells in one section was multiplied with the total number of sections. The number of TUJ1 positive cells was calculated using the same technique. The differentiation rate was estimated by dividing the number of differentiated cells by the number of surviving cells per specimen. All data are presented as the mean of each experimental group.

### 2.11. Statistics

Two-way ANOVA followed by Tukey's HSD test was used to determine statistically significant differences in cell numbers between the groups of animals. All results are expressed as mean ± standard error of the mean (SEM).

## 3. Results

The results described include four different animal groups (three or four rats in each group) shown in Table 1. In two groups, the survival time was three weeks and in one group it was six weeks. All groups were subject to the same surgical approach with HNPC cell injections to the AN by the IAM (Figure 1). The cells were harvested and processed from forebrain tissue obtained from a nine-week-old (postconception) human embryo. In all cell injection groups the BDNF was applied in the PA gel previously described. One control group did not receive any cell injections or BDNF. Immunohistochemistry and staining were used to identify transplanted cells and to detect possible differentiation. Cells double labeled (yellow) with GFP (green) and TUJ1 (red) were quantified as differentiated cells. The results are presented as comparisons between the groups with respect to cell survival and migration patterns as well as cell differentiation. The results of the cell quantification are summarized in Figure 5. As no cells were found in the modiolus or in the ST, these regions are not further mentioned in this section.

### 3.1. Higher Numbers of GFP+ HNPCs after BDNF-Treatment

Quantification of the number of GFP+ cells includes both the GFP+ HNPCs with immature profiles and GFP+ HNPCs differentiated into TUJ1 positive cells (Figure 5(a)).

**Figure 2:**
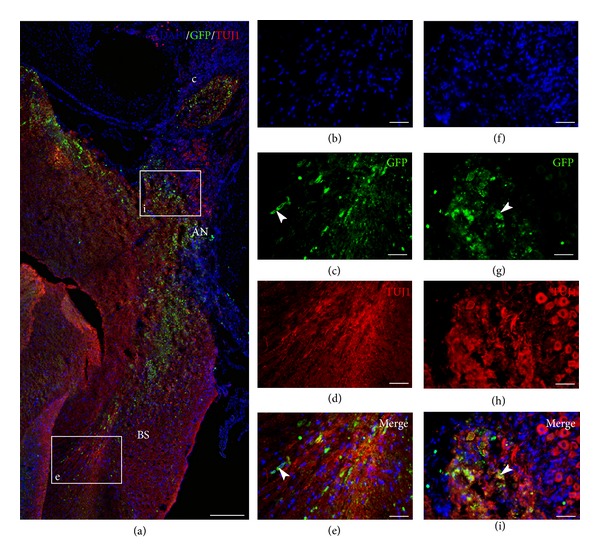
GFP+ HNPCs injected to the internal auditory meatus with application of PA gel only (Group 1). Immunohistochemical staining was performed with DAPI ((b), (f)) for cell nuclei, GFP ((c), (g)) for transplanted cells, and TUJ1 ((d), (h)) to verify differentiation. At three weeks following transplantation, GFP positive cells were identified along the auditory nerve “i” and in the brain stem “e.” Only a few cells have differentiated as verified by yellow double staining (GFP and TUJ1; (a), (c), (d), (e), (g), (h), and (i)). White arrowheads indicating surviving GFP+ HNPCs in the auditory nerve “i” and in the BS “e.” AN: auditory nerve; BS: brain stem; C: cochlea. Scale bar (a) 400 *μ*m, (b–i) 100 *μ*m.

**Figure 3:**
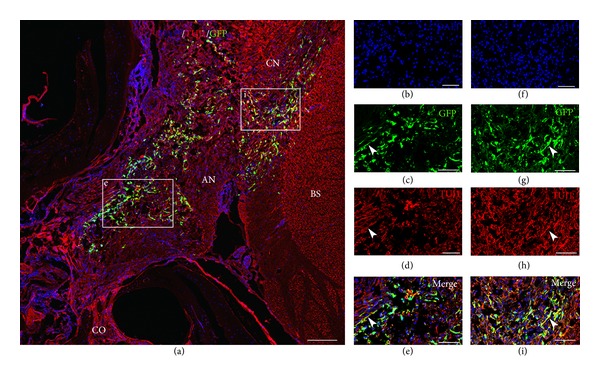
GFP+ HNPCs three weeks after transplantation with BDNF in PA gel (Group 2). Immunohistochemical staining was performed with DAPI ((b), (f)) for cell nuclei, GFP ((c), (g)) for transplanted cells, and TUJ1 ((d), (h)) to verify differentiation. Transplanted HNPCs were identified along the auditory nerve “e” and had also migrated to the brain stem “i.” Double-labeled yellow cells (GFP and TUJ1) and neurites (arrowheads) indicate neuronal differentiation ((a), (c), (d), (e), (g), (h), and (i)). CO: cochlea; AN: auditory nerve; BS: brain stem; CN: cochlear nucleus. Scale bar (a) 200 *μ*m, (b–i) 100 *μ*m.

**Figure 4:**
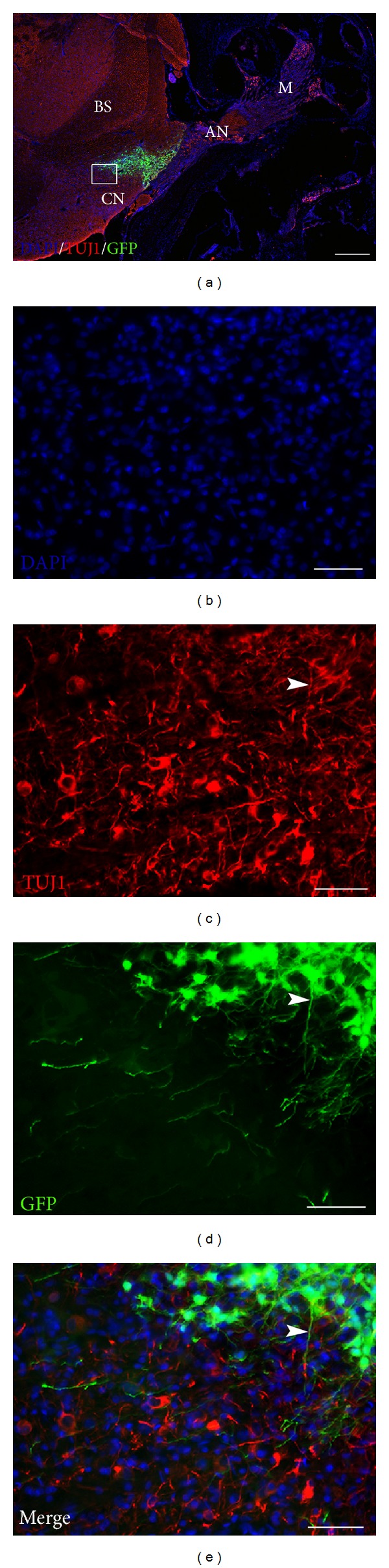
GFP+ HNPCs six weeks after transplantation with BDNF in PA gel (Group 3) (a). Cryostat section of dissected rat cochlea, auditory nerve, and brain stem following transplantation of HNPCs. Inset box in (a) shown at higher magnification in ((b)–(e)). Immunohistochemical staining was performed with DAPI (b) for cell nuclei, GFP (d) for transplanted cells, and TUJ1 (c) to verify differentiation. Double labeling (yellow) of GFP and TUJ1 indicates neuronal differentiation of the transplanted HNPCs. Double-labeled GFP, TUJ1 positive cells, and their arborisation of fibers can be identified in vicinity of the cochlear nucleus ((c)–(e)); arrowhead indicates GFP/TUJ1 double-labeled fiber. M: modiolus; AN: auditory nerve; BS: brain stem; CN: cochlear nucleus. Scale bar (a) 400 *μ*m, ((b)–(e)) 50 *μ*m.

**Figure 5:**
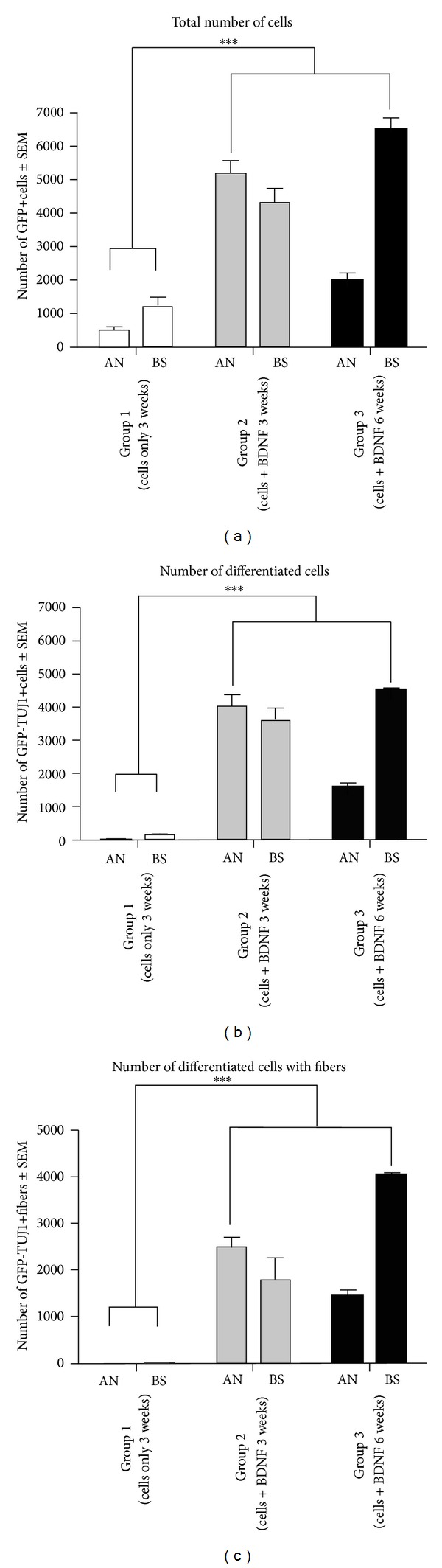
Graphs illustrating the quantification of numbers of GFP+ HNPCs, differentiated cells, and cells with neuronal outgrowths. (a) Total number of GFP+ HNPCs. Groups 2 and 3 had significantly better cell survival compared to Group 1 (***P ≤ 0.001). After 3 weeks the majority of the surviving cells were observed in the AN whereas after 6 weeks the majority of the cells were found in the BS. No cells were found in Group 4 (data not shown). (b) Number of double-labeled (GFP and TUJ1) differentiated cells. Groups 2 and 3 had significantly more differentiated cells than Group 1 (***P ≤ 0.001). After six weeks, the majority of the differentiated cells were found in the BS. (c) Number of differentiated cells with outgrowth of fibers. Groups 2 and 3 had significantly more cells with fiber outgrowth as compared to Group 1 (***P ≤ 0.001).

Surviving GFP+ HNPCs were found in all grafted animals. Overall, Groups 2 and 3 (both receiving BDNF-treatment) had significantly higher numbers of GFP+ cells (9562 ± 699 and 8489 ± 561, resp.) as compared to Group 1, which did not receive BDNF-treatment (1699 ± 383; *P* < 0.001; Figure 5(a), cf. Figures 2–4). When the regions were quantified separately, Groups 2 and 3 displayed a significantly higher number of GFP+ cells in both the AN and the BS (Group 2: 5196 ± 375 (AN); 4366 ± 08 (BS), Group 3: 1989 ± 226 (AN); 6500 ± 362 (BS)) as compared to Group 1 (481 ± 112 (AN), 1217 ± 274 (BS); Figure 5(a), cf. Figures 3 and 4). In Group 3 a majority of GFP+ cells were found in the BS as compared to Group 2, where the majority of GFP+ cells were found in the AN (Figure 5(a), cf. Figures 3 and 4).

### 3.2. BDNF in the PA Gel Promotes HNPC Differentiation

Transplanted cells were considered to have differentiated into a neuronal lineage if these were double stained with GFP and TUJ1. Overall, Groups 2 and 3 displayed significantly more neuronal differentiated cells (7590 ± 639 and 6088 ± 84, resp.) as compared to Group 1 (147 ± 71; Figure 5(b), cf. Figures 2–4). When the regions were analyzed separately, Groups 2 and 3 had significantly more differentiated cells in both the AN and the BS (Group 2: 4002 ± 359 (AN); 3588 ± 381 (BS), Group 3: 1577 ± 137 (AN); 4511 ± 57 (BS)) as compared to Group 1 (22 ± 22 (AN), 126 ± 50 (BS); *P* < 0.001; Figure 5(b), cf. Figures 3 and 4). Thus, out of the GFP+ HNPCs the most prominent neuronal differentiation was found in Groups 2 (79%) and 3 (72%). There were similar numbers of differentiated cells in the BS in Groups 2 and 3 (Figure 5(b)) but significantly more cells in the AN in Group 2 as compared to Group 3 (Figure 5(b); *P* < 0.001).

### 3.3. BDNF in the PA Gel Promotes Neuronal Outgrowths/Extensions in Differentiated Cells

Quantification of numbers of GFP+ HNPC with neuronal outgrowths was performed as described above. These were defined as GFP and TUJ1 positive cells that had outgrowths that were estimated to be at least equal in length to the cell soma. Overall, Groups 2 and 3 had significantly more cells with neuronal outgrowth (4240 ± 474 and 5473 ± 87, resp.; Figure 5(c)) as compared to Group 1 (17 ± 4; *P* < 0.001). When we analyzed each region individually we found that there were zero cells with outgrowths in the AN and only 17 ± 4 in the BS in Group 1 (Figure 5(c)). Group 2 had approximately an equal number of cells with outgrowths in the AN (2454 ± 237) as in the BS (1786 ±446). However, after 6 weeks of survival (Group 3), there were 4030 ± 33 cells with outgrowth in the BS as compared to 1443 ± 104 in the AN. This is illustrated in Figure 5(c).

## 4. Discussion

Here we present a novel nerve regeneration protocol for HNPC transplantation into a damaged rat AN with coapplication of PA gel and BDNF. In the present study, surviving and differentiated GFP+ HNPCs were detected in all groups in both the AN and the BS for up to six weeks after transplantation into the *β*-BuTx-damaged rat AN. At six weeks after transplantation, a higher number of GFP+ HNPCs were found in the BS as compared to the number of cells at three weeks, demonstrating central-directed (from the C.Inj. site) cell migration over time. Additionally, significantly more fiber outgrowths were seen at the six-week time point, demonstrating a progressive neuronal maturation over time of the grafted HNPC.

Further, the results indicate that BDNF had a significant effect on numbers of GFP+ cells and differentiation of the transplanted HNPCs. BDNF was mixed and applied in a PA gel over the injection site. This neurite-promoting laminin epitope containing gel has in several studies been shown to promote the regeneration of sensory nerve fibers, to increase survival and differentiation of stem cells and neural progenitors, and to have a suppressing effect on astrogliosis [28, 36]. This gel is intended to be mixed with transplanted cells and applied directly to the nerve lesion. However, due to limited space in the AN trunk in our experimental setup, we were unable to use the gel as intended but instead we applied the gel mixed with BDNF on the C.Inj. site. Although this application approach of the gel may not have been ideal, it is possible that our cell transplantation strategy benefited from the inhibitory effect of the PA gel on the glial scarring that can occur around the injection site, thus allowing for an increased migration of transplanted cells. Further, the cylindrical and hydrated nanofibers in the PA gel may bind and localize neurotrophic factor protecting it from enzymatic degradation. The gel network may also allow for sustained release of BDNF over time. Recently Angeloni and coworkers, using a different PA gel, demonstrated the possibility of regenerating the cavernous nerve by delivering Sonic hedgehog from a PA gel but not when the same morphogen was encapsulated in conventional gel beads [37].

To evaluate the effect of BDNF on the numbers of GFP+ cells, differentiation, and migration of the transplanted HNPCs, we quantified the number of GFP+ cells (Figure 5(a)) as well as the number of differentiated GFP and TUJ1 positive cells in the AN and BS (Figure 5(b)). Further we evaluated the number of differentiated GFP and TUJ1 positive cells with fiber outgrowth (Figure 5(c)). Branching is a necessary feature for axons in the development of complex neuronal circuits [38]. Studies of the retinotectal system have illustrated that BDNF, through activation of the TrkB, promotes branching of retinal axons [38, 39]. Further, the arborisation of neurite processes in the period of neurite outgrowth adds to the complexity of neuronal circuits [40]. Later retraction and pruning of inappropriate branches result in a more mature pattern of connectivity [41].

Regeneration of a sensory cranial nerve such as the AN presents several obstacles. First, cells suitable for the task need to be selected. There are several cell candidates in various stages of differentiation to choose from. Most favorable would be to transplant autogenic cells as this circumvents the issue with immunogenic responses and host versus graft rejection. Since the eventual goal of our experiments is to develop a cell replacement strategy that can be used clinically, we presently used HNPCs as cell candidate. In contrast to rodent stem cells, HNPCs have the potential to be used as a future allogeneic cell replacement therapy in humans. The HNPCs were initially obtained from first-trimester human embryonic forebrain tissue and can be long term expanded into high numbers* in vitro* [42] maintaining the capacity to form neurons, astrocytes, or oligodendrocytes, the three main phenotypes in the CNS [32]. Second, there is the issue of surgical approach. The AN is embedded in the cochlea and the temporal bone. To access the distal part of the nerve, it is therefore necessary to perform a cochleostomy; that is, penetrate the basal turn of the cochlea and access the nerve via the modiolus. This approach may therefore jeopardize the integrity and homeostasis of the cochlea. However, the central portion of the AN near the IAM can be accessed via a craniotomy. Once the craniotomy is performed, the cerebellum is retracted medially to reveal the AN going into the IAM. In a previous experiment, we have demonstrated that this central approach does not significantly affect the hearing of the experimental animals as measured by auditory brain stem response testing [34]. Third, the injected cells need to differentiate into neurons and find the proper connections both peripherally with the hair cells and centrally with cochlear nucleus neurons. BDNF has been shown to promote the neural differentiation of stem cells [43–45]. BDNF has also been shown to promote axonal branching in retinal ganglion cells through activation of the TrkB receptor [38]. Since the transplanted cells may need support by exogenous factors to survive and differentiate, one impediment may be how to give the cells trophic support over time. This may be achieved by prolonged infusions via miniosmotic pumps or containers with slow releasing agents such as a PA gel. Indeed one* in vitro* study has demonstrated that BDNF tethered with a nanofiber scaffold enhanced the proliferation and differentiation of cultured NSCs as compared to soluble BDNF [44]. Here we used a PA gel mixed with BDNF in order to provide prolonged trophic support to the HNPCs.

Overall, we observed very high neuronal differentiation rates of the transplanted GFP+ HNPCs in the BDNF-treated groups. At 3 weeks, 79% of the GFP+ HNPCs were TUJ1 positive. After 6 weeks of survival 72% of the GFP+ HNPCs were TUJ1 positive, which may indicate good survival of the human neurons over time. Additionally, from the total number of differentiated cells we observed a higher rate of cells with fiber outgrowth at 6 weeks 90% as compared to cells with fiber outgrowths at 3 weeks 56% (Figure 5), in the BDNF-treated groups, indicating that these cells appear to be maturing over time. These effects on numbers of GFP+ cells, differentiation, and sprouting appear to be due to BDNF since significantly more GFP+ cells, neuronally differentiated cells, and sprouts were found in Groups 2 and 3 as compared to the untreated Group 1.

In the BDNF-treated groups, even though total GFP+ cell numbers were similar between the three and six weeks of survival groups, the locations of the surviving cells were different. The differentiated GFP+ cells at the 3-week time point were evenly distributed between the AN (54% GFP, 53% GFP/TUJ1) and the BS (46% GFP, 47% GFP/TUJ1). However, at the 6-week time point the majority of surviving and differentiated cells had migrated into the BS (77% GFP, 74% GFP/TUJ1) as compared to the AN (23% GFP, 26% GFP/TUJ1) indicating a central migration of the cells over time. The environment in the BS being more favorable for harboring the transplanted cells could explain this and that site-specific cues in the BS may trigger migration, differentiation, and sprouting of these cells.

Additionally, the number of double-stained cells with fiber outgrowths in the BDNF-treated groups was also significantly higher in the BS than in the AN after 6 weeks as compared to the 3-week group. Therefore, it appears that BDNF promoted differentiation and these differentiated cells developed fibers as the cells matured over time.

## 5. Conclusion

Regeneration of cranial nerves constitutes a major challenge both in the control of the microcellular environment and in getting the proper surgical access without damaging sensitive structures. The current study comprises a first step in finding novel therapeutic techniques for aiding patients with a severely damaged AN. As CIs and ABIs are getting more advanced, higher demands on the function of the residual AN and spiral ganglions are to be expected. A combination therapy with CI, cell transplantation, and neurotrophic factor stimulation may be a feasible approach. For a future clinical use, the cells need to be derived from human sources. In this study, we observed high survival and neuronal differentiation of HNPCs transplanted to the rat AN. Our results also indicate that HNPC integration with host tissue benefits from additional BDNF. As this study did not include verification of synapses or functional assessment, we cannot draw any conclusions on the integration of the transplanted cells into the existing neuronal circuitry. However, the fact that we found differentiated HNPCs and newly formed nerve fibers in close proximity to the cochlear nucleus suggests the need for further studies to explore the functional effectiveness of this cell transplantation paradigm.

## Figures and Tables

**Table 1:** Animal groups. Group 1 (*n* = 3; survival time 3 weeks) had HNPCs injected to the AN with PA gel applied on the injection site. Group 2 (*n* = 4; survival time 3 weeks) had HNPCs injected to the AN with PA gel including BDNF applied on the injection site. Group 3 (*n* = 3; survival time 6 weeks) had HNPCs injected to the AN with PA gel with BDNF applied on the injection site. Group 4 was the control group (*n* = 3; 3-week survival time) and had medium only without cells injected to the nerve.

Group	Number of animals	*β*-BuTx	HNPC injections	BDNF	PA gel	Survival time
1	3	+	+		+	3 weeks
2	4	+	+	+	+	3 weeks
3	3	+	+	+	+	6 weeks
4	3	+				3 weeks

*β*-BuTx, *β*-bungarotoxin; HNPC, human neural precursor cells; BDNF, brain derived neurotrophic factor; PA, peptide amphiphile.
